# A guide to cancer genetics in clinical practice

**DOI:** 10.1038/sj.bjc.6605693

**Published:** 2010-06-08

**Authors:** L Side

**Affiliations:** 1 Gynaecological Cancer Research Centre, Institute for Women's Health, UCL, 149 Tottenham Court Road, London W1T 7DN, UK

It is now widely recognised in clinical practice that many of the common cancers have a heritable component. With advances in genetic technology and increasing awareness among the public, who may then seek genetic advice, more families with a cancer predisposition are being identified. Care of individuals from these families requires a co-ordinated multidisciplinary approach, not merely involving specialised clinical genetics services. In particular, screening and surgical services have an increasingly important role in their management. In the more common forms of familial cancer, such as hereditary breast and ovarian cancer, and Lynch syndrome (hereditary non-polyposis colorectal cancer), it is important that the generalist has a working knowledge of these conditions. This handbook aims to address the knowledge gap for those clinicians who may encounter and care for families with cancer predispositions as part of their everyday practice, but have no formal genetics training.

The editor is a consultant colorectal surgeon who is well known for her work with polyposis families. It is a multi-authored volume and the chapter authors are likewise recognised in their fields. Nevertheless, the overall bias of the book does reflect the editor's background. Of the 10 disease-specific chapters, 3 relate to polyposis syndromes and 6 to inherited cancers of the gastrointestinal tract. There are some notable omissions. For instance, there is a reference to Birt Hogg Dube syndrome in the introductory chapter, but inherited kidney cancer (and in particular von Hippel Lindau syndrome) is not covered. Apart from the chapter on neurofibromatosis, skin tumour predisposition syndromes are not specifically addressed. Multiple endocrine neoplasia syndromes are discussed, but not the more recent genetic knowledge about non-syndromic phaeochromocytoma. There is a historical reference to retinoblastoma, the paradigm for inherited cancers due to tumour suppressor genes, in Chapter 2, but other childhood cancers do not feature. This is perhaps to be expected in a volume of this size, aimed largely at clinicians in adult medicine. However, breast and gynaecological cancers have individual chapters devoted to them.

The introductory chapters aim to demystify genetics, and address modes of inheritance and some basic facts about DNA. The importance of taking a family history is emphasised, both at the beginning and at the end of the book. The third chapter deals with some of the difficulties encountered in looking after families, rather than treating individuals. Ethical dilemmas in genetics are covered with a useful series of practical examples, addressing issues such as the need for sensitivity and confidentiality in dealing with information when one cannot be certain how much of this has been disclosed within a family.

The chapters covering the different cancer syndromes are generally succinct and practical. There is a useful summary of key points at the end of each chapter. Illustrations are in black and white. Many of the chapters include tables summarising the main aspects of screening and management. In general, the authors provide a balanced view of the evidence for surveillance for different genetic conditions. However, some international differences are evident. For instance, in the chapter on familial pancreatic cancer (perhaps a surprising inclusion, as there is little available in terms of genetic testing as yet), the authors state that ‘there is general agreement that members of FPC kindreds should be offered screening’. This is not a universally held view; for example, in the United Kingdom, screening is generally offered in the context of a research study, as evidence that it improves outcome is currently lacking.

Some of the newer research trials are discussed, such as the aspirin chemoprevention study in Lynch syndrome (CAPP2) and PARP inhibitors in BRCA gene carriers. These interventions show promising results but have not yet been widely adopted into clinical practice. Although the majority of chapters address high-risk cancer genes, genome-wide association studies that identify common low penetrance variants are addressed in the breast cancer chapter and in the chapter on future developments. This is a burgeoning area of research likely to affect clinical practice in the future, so perhaps more space could have been devoted to it. Reproductive issues and more recent developments in this field, including pre-implantation genetic diagnosis for cancer predisposition genes, are discussed.

The book title promises that it is a guide to cancer genetics in clinical practice. However, somatic cancer genetics and genetic testing of tumours is not covered, which is perhaps a missed opportunity. Not only is this affecting the treatment of cancer, but may also uncover an unidentified tumour predisposition (as in the mismatch repair genes in Lynch syndrome). Nevertheless, the book is successful as a guide to inherited cancer syndromes for a broad clinical audience, and providing a basis for the management of these conditions. It is probably most useful for those in the field of gastroenterology and colorectal surgery, as this is the focus of many of the specialist chapters.

